# A rare occurrence of isolated superior mesenteric artery dissection, a case image report

**DOI:** 10.1002/ccr3.9121

**Published:** 2024-06-28

**Authors:** Nida Ansari, Sacide S. Ozgur, Alan Alcantara, Patrick Michael

**Affiliations:** ^1^ Department of Internal Medicine St. Joseph's University Medical Center Paterson New Jersey USA

**Keywords:** abdominal pain, dissection, isolated, spontaneous, super mesenteric artery

## Abstract

Isolated spontaneous superior mesenteric artery (SMA) dissection is relatively rare. Often found incidentally on cross‐sectional imaging, often managed non‐operatively. We present a patient who presented with chest pain and was found to have a SMA dissection.

## 1 CASE PRESENTATION

**FIGURE 1:**
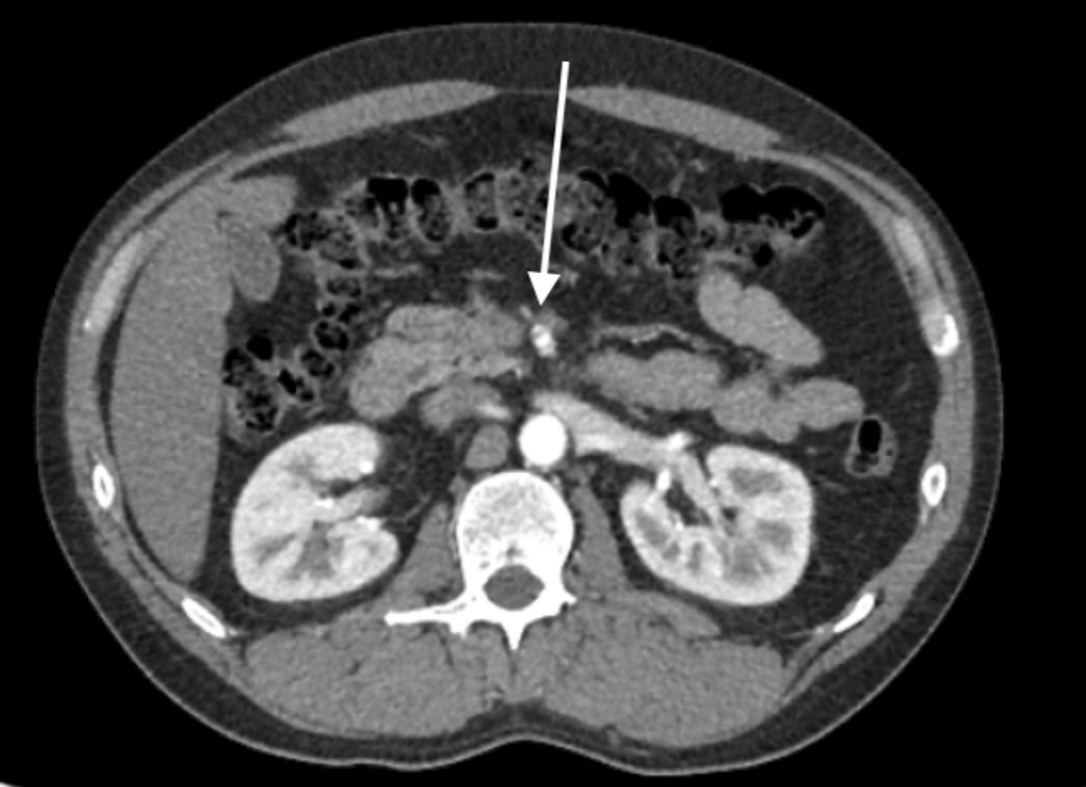
Axial view of computer tomography angiography of the chest, abdomen, and pelvis showing proximal superior mesenteric artery.

A 39‐year‐old male with a past medical history of alcohol abuse disorder presented to the emergency department (ED) with chest pain with radiation to the left arm. Vitals in the ED showed a heart rate of 91 bpm, blood pressure of 127/95 mm Hg, oxygen saturation of 100% on room air. EKG was obtained and showed a heart rate of 70 bpm and sinus rhythm with no ischemic changes. Troponin was sent which was negative. To evaluate further etiologies, computer tomography angiography of the chest, abdomen, and pelvis was performed, which revealed a small dissection of the proximal superior mesenteric artery (Figure 1). It was also noted that the thoracic and abdominal aorta were patent, as were the celiac, inferior mesenteric and bilateral renal arteries are patent to the extent visualized. Vascular surgery was consulted; however, no surgical intervention was performed as distal flow beyond the dissection was noted. Patient was placed on strict blood pressure control and given nitroglycerin as needed for chest pain. His symptoms ultimately resolved and he was discharged home.

### Discussion

Spontaneous idiopathic superior mesenteric artery dissection is rare and could be fatal by inducing bowel ischemia, infarction, and potentially death.^1^, ^2^ Patients typically present with an acute onset of abdominal pain, back pain, and flank pain however, patients can be asymptomatic.^2^ More often seen in men than women, risk factors include hypertension, atherosclerosis, connective tissue disorders, vasculitis, trauma, or idiopathic.^1^ The gold standard for diagnosis includes computer tomography or magnetic resonance angiography.^1^ In regards to management, surgical intervention, such as stenting, is warranted when there is concern for bowel ischemia or aneurysmal degeneration.^2^ In a study by Morgan et al., where 77 patients with spontaneous mesenteric dissection, only four required surgical intervention, and 73 were managed conservatively.^2^ This was the case for our patient.

## AUTHOR CONTRIBUTIONS

**Nida Ansari:** Conceptualization; data curation; formal analysis; funding acquisition; investigation; methodology; project administration; resources; software; supervision; validation; visualization; writing – original draft; writing – review and editing. **Sacide S. Ozgur:** Conceptualization; data curation; formal analysis; funding acquisition; investigation; methodology; project administration; resources; software; supervision; validation; visualization; writing – original draft; writing – review and editing. **Alan Alcantara:** Conceptualization; data curation; formal analysis; funding acquisition; investigation; methodology; project administration; resources; software; supervision; validation; visualization; writing – original draft; writing – review and editing. **Patrick Michael:** Conceptualization; data curation; formal analysis; funding acquisition; investigation; methodology; project administration; resources; software; supervision; validation; visualization; writing – review and editing.

## CONFLICT OF INTEREST STATEMENT

The authors report no conflict of interest. An ethical review is not necessary because this is a case report. This research received no specific grant from funding agencies in the public, commercial, or not‐for‐profit sectors.

## CONSENT

Written informed consent was obtained from the patient to publish this report in accordance with the journal's patient consent policy.

## Data Availability

Data availability is not applicable to this article as no new data were created or analyzed in this study.
